# Supplementary material for: Association between hot flashes severity and oxidative stress among Mexican postmenopausal women: A cross-sectional study
Source: PLoS One. 2019 Sep 24;14(9):e0214264. doi: 10.1371/journal.pone.0214264 (PMC6759180; doi:10.1371/journal.pone.0214264)
Supplement: S1 File — (DOCX) [file pone.0214264.s001.docx]

|  | |  |
| --- | --- | --- |
|  | FACULTAD DE ESTUDIOS SUPERIORES * Z A R A G O Z A *  UNIDAD DE INVESTIGACIÓN EN GERONTOLOGÍA  DESCRIPCIÓN DE BOCHORNOS | |

| Nivel de bochorno | Descripción |
| --- | --- |
| Leves | - **Duración**: menos de 5 minutos - **Síntomas físicos**: calor con sentimientos de incomodidad, cara roja. |
| Moderados | - **Duración:** hasta 15 minutos - **Síntomas físicos:** cabeza, cuello, orejas o el cuerpo entero se siente caliente; los músculos están tensos; algunas sudoraciones; sequedad de boca - **Síntomas emocionales:** irritabilidad, agitación, pérdida de energía, cansancio, sentimientos de vergüenza cuando se siente un bochorno enfrente de otros. |
| Severos | - **Duración:** hasta 20 minutos - **Síntomas físicos**: calor que se pueden describir como estar quemándose; un cambio en la frecuencia cardiaca; debilidad; dolor de cabeza; severas sudoraciones; sensaciones punzantes sobre la piel; pesadez en el pecho - **Síntomas emocionales:** vergüenza, ansiedad, sensación de un ataque de pánico. |
| Muy severos | - **Duración:** hasta 45 minutos - **Síntomas físicos:** temperatura muy elevada, sudoración continua, dificultades para respirar, debilidad, mareos, calambres en las piernas, un cambio en el ritmo cardiaco, sensación de estar enferma del estómago - **Síntomas emocionales:** angustia, sensación de querer escapar, dificultad para realizar las actividades |

**HOT FLASHES DESCRIPTION**

| Hot flash level | Description |
| --- | --- |
| Mild | - **Duration**: less 5 minutes - **Physical symptoms**: heat with feelings of discomfort, red face. |
| Moderate | - **Duration:** up to 15 minutes - **Physical symptoms:** head, neck, ears or the whole body feels hot; the muscles are tense; some sweats; dry mouth - **Emotional symptoms:** irritability, agitation, loss of energy, tiredness, feelings of shame when you feel an embarrassment in front of others. |
| Severe | - **Duration:** up to 20 minutes - **Physical symptoms**: heat that can be described as burning; a change in heart rate; weakness; headache; severe sweating; stabbing sensations on the skin; heaviness in the chest - **Emotional symptoms:** shame, anxiety, feeling of a panic attack |
| Very severe | - **Duration:** up to 45 minutes - **Physical symptoms:** very high temperature, continuous sweating, breathing difficulties, weakness, dizziness, leg cramps, a change in heart rhythm, feeling sick to the stomach - **Emotional symptoms:** angst, feeling of wanting to escape, difficulty performing activities |
