# Supplementary material for: Association between hot flashes severity and oxidative stress among Mexican postmenopausal women: A cross-sectional study
Source: PLoS One. 2019 Sep 24;14(9):e0214264. doi: 10.1371/journal.pone.0214264 (PMC6759180; doi:10.1371/journal.pone.0214264)
Supplement: S2 File — (DOC) [file pone.0214264.s002.doc]

|  | FACULTAD DE ESTUDIOS SUPERIORES * Z A R A G O Z A * **2017** UNIDAD DE INVESTIGACIÓN EN GERONTOLOGÍAESCALA DE ANSIEDAD DE ZUNG Clave: (ASI) |
| --- | --- |

Nombre: _______________________________________________________­­­­­­­­­­­­­­­­____________________________________

Edad: __________ Sexo: _________ Fecha de evaluación: _____________________

**INSTRUCCIONES:** Esta escala está diseñada para la detección y medida de la gravedad de la ansiedad. Por favor marque con una cruz (X), en el cuadro correspondiente a la intensidad, duración y frecuencia de los síntomas señalados en la última semana.

|  |  | Ausente  1 | Ligero  2 | Moderado  3 | Intenso  4 |
| --- | --- | --- | --- | --- | --- |
| 1 | ¿Se siente nervioso, ansioso? |  |  |  |  |
| 2 | ¿Se siente asustado? |  |  |  |  |
| 3 | ¿Se aterroriza con facilidad? |  |  |  |  |
| 4 | ¿Se siente como si fuera a volverse loco? |  |  |  |  |
| 5 | ¿Se siente como si fuera a ocurrir algo terrible? |  |  |  |  |
| 6 | ¿Se siente tembloroso? |  |  |  |  |
| 7 | ¿Tiene dolores de cabeza, cuello o espalda? |  |  |  |  |
| 8 | ¿Se cansa con facilidad? ¿se siente débil a ratos? |  |  |  |  |
| 9 | ¿Se encuentra inquieto? ¿hasta el punto de no poder estar sentado? |  |  |  |  |
| 10 | ¿Siente que su corazón late de prisa? |  |  |  |  |
| 11 | ¿Se siente mareado a ratos? |  |  |  |  |
| 12 | ¿Se ha desmayado a veces? ¿ha sentido como si fuera a desmayarse? |  |  |  |  |
|  |  | Ausente  1 | Ligero  2 | Moderado  3 | Intenso  4 |
| 13 | ¿Siente dificultad para respirar? |  |  |  |  |
| 14 | ¿Tiene sensación de adormecimiento en los dedos o alrededor de la boca? |  |  |  |  |
| 15 | ¿Siente náusea? ¿tiene vómito? |  |  |  |  |
| 16 | ¿Necesita ir a orinar con mucha frecuencia? |  |  |  |  |
| 17 | ¿Se siente sudoroso, con las manos húmedas y frías? |  |  |  |  |
| 18 | ¿Tiene bochornos? |  |  |  |  |
| 19 | ¿Le cuesta trabajo dormirse? |  |  |  |  |
| 20 | ¿Tiene sueños que le asustan, pesadillas? |  |  |  |  |
|  | Puntuación total |  |  |  |  |

Zung WW. A rating instrument for anxiety disorders. Psychosomatics. 1971; 12: 371-9.

Evaluador(a): __________________________________

Supervisor(a): __________________________________
