# Supplementary material for: Association between hot flashes severity and oxidative stress among Mexican postmenopausal women: A cross-sectional study
Source: PLoS One. 2019 Sep 24;14(9):e0214264. doi: 10.1371/journal.pone.0214264 (PMC6759180; doi:10.1371/journal.pone.0214264)
Supplement: S3 File — (DOC) [file pone.0214264.s003.doc]

|  | FACULTAD DE ESTUDIOS SUPERIORES * Z A R A G O Z A * **2017** UNIDAD DE INVESTIGACIÓN EN GERONTOLOGÍAESCALA DE DEPRESIÓN DE ZUNG Clave: (SDS) |
| --- | --- |

Nombre: _______________________________________________________­­­­­­­­­­­­­­­­____________________________________

Edad: __________ Sexo: _________ Fecha de evaluación: _____________________

**INSTRUCCIONES:** Esta escala está diseñada para la detección y medida de la gravedad de la depresión. Por favor marque con una cruz (X), en el cuadro correspondiente a la opción seleccionada que indique cómo se ha encontrado en el momento actual o recientemente. Sólo podrá emitir una respuesta por cada una.

|  |  | Muy poco tiempo/ Muy pocas veces/ Raramente | Algún tiempo/ Algunas veces/ De vez en cuando | Gran parte del tiempo/ Muchas veces/ Frecuentemente | Casi siempre/ Siempre/ Casi toda el tiempo |
| --- | --- | --- | --- | --- | --- |
| 1 | Me siento triste y deprimido(a). |  |  |  |  |
| 2 | Por las mañanas me siento peor que por las tardes. |  |  |  |  |
| 3 | Frecuentemente tengo ganas de llorar y a veces lloro. |  |  |  |  |
| 4 | Me cuesta mucho dormir o duermo mal por la noche. |  |  |  |  |
| 5 | Ahora tengo menos apetito que antes. |  |  |  |  |
| 6 | Me siento menos atraído(a) por el sexo opuesto |  |  |  |  |
| 7 | Creo que estoy adelgazando. |  |  |  |  |
| 8 | Estoy estreñido(a) (constipado(a)). |  |  |  |  |
| 9 | Tengo palpitaciones. |  |  |  |  |
| 10 | Me canso por cualquier cosa. |  |  |  |  |
| 11 | Mi cabeza no está tan despejada como antes. |  |  |  |  |
|  |  | Muy poco tiempo/ Muy pocas veces/ Raramente | Algún tiempo/ Algunas veces/ De vez en cuando | Gran parte del tiempo/ Muchas veces/ Frecuentemente | Casi siempre/ Siempre/ Casi toda el tiempo |
| 12 | No hago las cosas con la misma facilidad que antes |  |  |  |  |
| 13 | Me siento agitado(a) e intranquilo(a) y no puedo estar quieto(a). |  |  |  |  |
| 14 | No tengo esperanza y confianza en el futuro. |  |  |  |  |
| 15 | Me siento más irritable que habitualmente. |  |  |  |  |
| 16 | Encuentro difícil la toma de decisiones. |  |  |  |  |
| 17 | No me creo útil y necesario(a) para la gente. |  |  |  |  |
| 18 | No encuentro agradable vivir, mi vida no es plena. |  |  |  |  |
| 19 | Creo que sería mejor para los demás que me muriera. |  |  |  |  |
| 20 | No me gustan las mismas cosa que habitualmente me agradaban. |  |  |  |  |
|  | Puntuación total |  |  |  |  |
|  | Puntuación normalizada |  |  |  |  |

Zung WW. A self rating depression scale. Arch Gen Psychiatr. 1965; 12: 63-70.

Evaluador(a): __________________________________

Supervisor(a): __________________________________
