# Supplementary material for: Association between hot flashes severity and oxidative stress among Mexican postmenopausal women: A cross-sectional study
Source: PLoS One. 2019 Sep 24;14(9):e0214264. doi: 10.1371/journal.pone.0214264 (PMC6759180; doi:10.1371/journal.pone.0214264)
Supplement: S4 File — (DOC) [file pone.0214264.s004.doc]

|  | FACULTAD DE ESTUDIOS SUPERIORES * Z A R A G O Z A * **2017** UNIDAD DE INVESTIGACIÓN EN GERONTOLOGÍAESCALA ATENAS DE INSOMNIO Clave: |
| --- | --- |

Nombre: ______________________________________________________________

Edad: __________ Sexo: _________ Fecha de evaluación: _____________________

**INSTRUCCIONES:** Esta escala está diseñada para registrar su propia percepción de cualquier dificultad en el dormir que usted pudiera haber experimentado. Por favor marque (encerrando en un círculo el número correspondiente) la opción debajo de cada enunciado para indicar su estimación de cualquier dificultad, siempre que haya ocurrido durante la última semana.

1. **Inducción del dormir** (tiempo que le toma quedarse dormido una vez acostado).

| Ningún problema.  0 | Ligeramente retrasado.  1 | Marcadamente retrasado  2 | Muy retrasado o no durmió en absoluto.  3 |
| --- | --- | --- | --- |

1. Despertares durante la noche.

| Ningún problema.  0 | Problema menor  1 | Problema considerable  2 | Muy retrasado o no durmió en absoluto.  3 |
| --- | --- | --- | --- |

1. Despertar final más temprano de lo deseado.

| No más temprano  0 | Un poco más temprano  1 | Marcadamente más temprano  2 | Mucho más temprano o no durmió en lo absoluto  3 |
| --- | --- | --- | --- |

1. Duración total del dormir.

| Suficiente  0 | Ligeramente insuficiente  1 | Marcadamente insuficiente  2 | Muy insuficiente o no durmió en lo absoluto  3 |
| --- | --- | --- | --- |

1. **Calidad general del dormir** (no importa cuánto tiempo durmió usted).

| Satisfactoria  0 | Ligeramente insatisfactoria  1 | Marcadamente insatisfactoria  2 | Muy insatisfactoria o no durmió en lo absoluto  3 |
| --- | --- | --- | --- |

1. Sensación de bienestar durante el día.

| Normal  0 | Ligeramente  disminuida  1 | Marcadamente disminuida  2 | Muy  disminuida  3 |
| --- | --- | --- | --- |

1. Funcionamiento (físico y mental) durante el día.

| Normal  0 | Ligeramente  disminuida  1 | Marcadamente disminuida  2 | Muy  disminuida  3 |
| --- | --- | --- | --- |

1. Somnolencia durante el día.

| Ninguna  0 | Leve  1 | Considerable  2 | Intensa  3 |
| --- | --- | --- | --- |

Soldatos C, Dikeos D, Paparrigopoulos T. Athens Insomnia Scale: validation of an instrument based on ICD-10 criteria. J Psychosom Res. 2000; 48: 555-60.
